# Dynamic Rotational Sensor Using Polymer Optical Fiber for Robot Movement Assessment Based on Intensity Variation

**DOI:** 10.3390/polym14235167

**Published:** 2022-11-28

**Authors:** Jianwei Shi, Abdul Ghaffar, Yongwei Li, Irfan Mehdi, Rehan Mehdi, Fayaz A. Soomro, Sadam Hussain, Mujahid Mehdi, Qiang Li, Zhiqiang Li

**Affiliations:** 1 Department of Automation, Taiyuan Institute of Technology, Taiyuan 030051, China; 2 State Key Laboratory of Geomechanics and Geotechnical Engineering, Institute of Rock and Soil Mechanics, Chinese Academy of Sciences, Wuhan 430071, China; 3 University of Chinese Academy of Sciences, Beijing 100049, China; 4 Science and Technology on Electronic Test & Measurement Laboratory, North University of China, Taiyuan 030051, China; 5 Department of Chemical Engineering, QUEST Nawabshah, Nawabshah 67450, Pakistan; 6 National Centre of Excellence in Analytical Chemistry, University of Sindh, Jamshoro 76060, Pakistan; 7 Department of Management Science, Qurtuba University of Science and Information Technology, Peshawar 24800, Pakistan; 8 Key Laboratory of Air-Driven Equipment Technology of Zhejiang Province, College of Mechanical Engineering, Quzhou University, Quzhou 324000, China; 9 Institute of Turbomachinery, Xi’an Jiatong University, Xi’an 710049, China

**Keywords:** optical fiber sensor, polymer fiber, robotic movement assessment, dynamic rotation

## Abstract

A complex signal processing technique is usually required to process the data in most sensor design structures, and integration into real applications is also challenging. This work presents a dynamic rotational sensor using polymethyl methacrylate (PMMA) fiber for robot movement assessment. The sensor design structure is based on the coupling of light intensity, in which two PMMA fibers are twisted together. Both fibers are bent after twisting and attached on the linear translation stage, which is further attached to the robot. The variation in bending radius causes the bending loss, and that loss is coupled in the second fiber. The change in the macro-bend radius corresponds to the rotation of the robot. Experimental results indicate that the sensor can operate in full rotational cycle (i.e., 0°–360°) as well as for clock and anti-clockwise rotation. Moreover, different rotational speeds (2°/s, 3°/s, 5°/s, and 10°/s) were carried out. The hysteresis loss of the sensor was about 0.77% and the sensitivity was 8.69 nW/°. The presented dynamic rotational sensor is cost-effective and easily integrated into the robot structure to analyze the robot’s circular motion.

## 1. Introduction

In the commercial industry, users demand technology integration, which covers the product lifetime, maintains and simplifies the operation, and combines to ensure integrity and security. Furniture technology must also be adapted to quantitative, cost-effective, decisive, and control. For illustration, to detect the measurement of shaft movement (such as those found at the steering wheel corners of automatic, aero-engine, or similar vehicle engines), the shaft’s operating characteristics must be known to ensure the safe operation of any shaft system. A few essential operating functions include monitoring the lateral shaft offset, shaft speed, and torque. Currently, it is difficult to capture or detect all of these features for sensors using non-contact technology. At present, a number of different kinds of sensors are available for measuring single functions such as temperature [1], displacement [2,3], liquid [4], humidity [5], force [6], and sensor deformation [7]. Among them, the rotational sensor is vital in various technical solutions and has become the main object of research and development [8,9,10]. The importance of a rotational sensor has been applied in many applications such as the analysis the seismicity [11], as inclinometers in geotechnical engineering [12], the precise landing of unmanned aerial vehicle (UAV) [13], the control of the aircraft flight direction, the launch of weapons, the measurement structure of sizeable civil aircraft, and test models of wind tunnels [14,15,16,17]. Separate specifications require different expectations: some desired repeatability, some required accuracy and precise control of the circuit speed, high resolution, or low cycle error [18]. Optimal control is essential in machines with switchable resistors for the pulse excitation phase to generate the rotor angle information [19]. In contrast, the rotor’s position is measured directly by mechanical means such as a flashlight with a serrated driver or a Hall effect sensor [20]. Some rotational sensors are used for precision purposes such as shape measurement, surface mounting, and plate machining [21,22]. In addition, rotational sensors are commonly used in the automotive industry, gas turbines, and robotic movement.

A robot system navigates the physical world using three types of movement: linear, joint, and circular [23,24,25]. An arc or circular movement moves the robot tool frame around a constant circle. The robot’s path along the way is the primary determining factor for each type of move, even though the goal of each is to get from point A to B. A sensing technique could be used to detect and predict the robot’s point or location, which gives an accurate assessment of the robot position [26], while some other kinds of sensors are also used to detect different kinds of objects such as fruit [27,28,29,30]. Rotation is usually common in robotics, and physical sensors are used to track the rotation [31,32]. Furthermore, measuring and analyzing joint angles can be beneficial to clinicians and therapists, as it is used for evaluating and quantifying rehabilitation exercises and surgical interventions [33]. In addition to training athletes, kinematic measurements are used to control neural prostheses by using kinematic data [34,35].

However, the above rotational sensors are based on the conventional sensing method. The alternative approach to the developed rotational sensor is based on fiber-optics [36,37,38,39,40]. Among the different sensor designs, Azmi et al. [41] presented a rotational and dynamic bending sensor using seven core fibers based on coherence interferometry, where they achieved a sensitivity of 6.2 fringe/° and the range was 360°. However, the fiber-based rotational sensors were developed using either glass optical fibers (GOFs) or polymer optical fibers (POFs) [38,42]. The POFs have unique characteristics over GOFs as POFs are cheap, flexible, and easy to handle as well as easy to bend [43]. Based on POFs, Leal et al. [44] proposed a curvature sensor using POF and achieved rotation up to 90°. However, this method has not achieved the fiber breakage anxiety and full rotational cycle response. Demetrio et al. [45] also presented a POF-based rotational sensor employing a three-loop fiber. He used a custom-made extrusion die to fabricate the PMMA fiber, but the measurement range was limited up to 50°.

Our goal was to present a simple, low-cost, and easy-to-build method for dynamic rotation measurement sensors that should be easy to integrate into the robot structure. The sensor was based on twisting two fibers and coupling the intensity of the macro-bend loss. The work was divided into two parts: designing the sensor and implementing the designed sensor on the robot structure. The power meter, light source, and POF pair were used to design the sensor structure. Then, the sensor was attached to the robot structure and the robot’s movement pulls the twisted fibers, resulting in a bend–radius change. A bending loss occurs in the transmitting fiber and couples in the second fiber, with the coupling power corresponding to different rotational positions. The details of the design sensor structure and implementation of the robot structure are given in the following sections.

## 2. Materials and Methods

**Figure 1:**
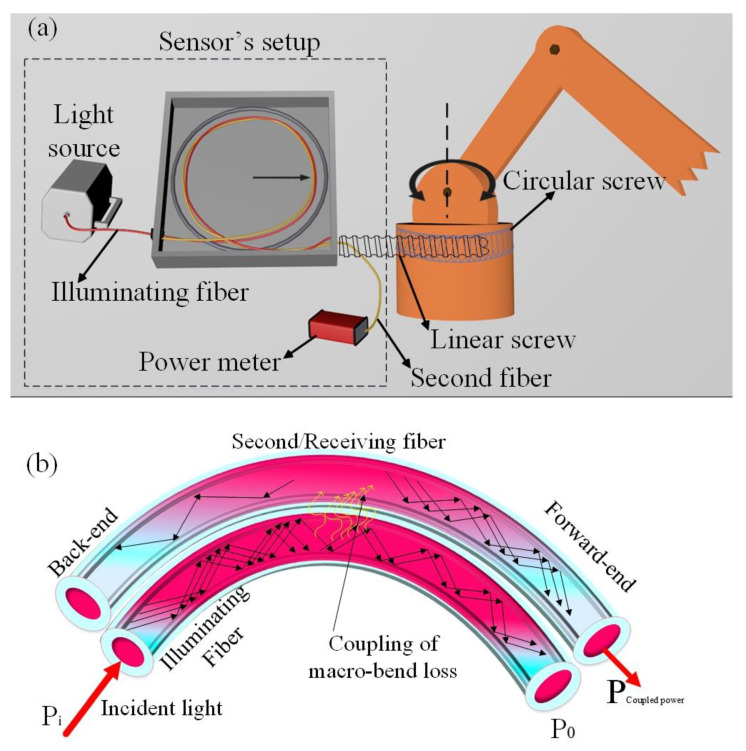
Schematic diagram of the rotational sensor. (**a**) The sensor is integrated into a robot for the rotational movement assessment, (**b**) the schematic diagram of the sensor’s working principle.

The dynamic rotational sensor design setup includes the polymethyl methacrylate (PMMA) fiber (Mitsubishi’s SK-40, Tokyo, Japan), a light source (fiber-coupled LED M660F1, Thorlabs), a power-meter (PM100USB, Thorlabs containing S151c photodetector), and a robot. The PMMA fiber’s characteristic parameters are given in Table 1. The resolution of the power meter is 1 pW. The robot’s movement is based on a stepper motor and is to operate the system at various rotational positions. The schematic diagram of the structure is shown in Figure 1a.

In the sensor design arrangement, two uncoated POFs were twisted together (the sensor parameters are given in Table 2. The length of uncoated fiber was 1 m, and the twisting length was 10 cm. After twisting, a black tube was used to coat the fiber to shield the external light [46]. The light source was a LED with a wavelength of 660 nm, and the initial power was 10 mW. The light source was coupled with the first POF termed as the illuminating fiber. In contrast, the second POF was not connected with any laminate source. It is a well-known fact that light can be coupled from one fiber to another if two fibers are placed close to each other, which is known as couple-mode theory [47]. The light propagating in the second optical fiber is based on couple-mode theory, where the distorted macro-bending loss is coupled in the second fiber. The bending loss is radiated from the illumination fiber, which is coupled to the second fiber and detected through the power meter. Figure 1b shows the schematic diagram for coupling the macro-bend loss from the illuminating fiber into the second fiber. The coupled macro-bend loss power in the second fiber can be calculated by Equation (1) [47].

(1) $$P_{c}=\sqrt{1+{\left(\frac{C}{\sqrt{\mathrm{nC}}}\right)}^{2}}$$

where C is the coupling coefficient between two fibers; $P_{c}$ is the coupled power in the second fiber. Usually, the macro-bend loss is being coupled, while the coupling coefficient C can be estimated by Equation (2);

(2) $$C=\frac{\sqrt{\delta }U^{2}K_{0}\left[W\left(\frac{d}{\rho }\right)\right]}{V^{3}K_{1}^{2}\left(W\right)}$$

(3) $$V=U^{2}+{\;W}^{2}$$

(4) $$\delta =1-{\left(\frac{n_{2}}{n_{1}}\right)}^{2}\;$$

where V represents the dimensionless frequency; ρ is the core radius; the degree of isolation between two fibers can be determined by $W\left(\frac{d}{\rho }\right)$. K’s is the modified Hankel functions, $n_{1}$ and $n_{2}$ are the refractive index of the core and cladding, respectively. In the coupled-mode theory, the distance between two fibers is essential, represented as d. The other important factor is the coupling length, and this is given by Equation (5):

(5) $$l=\frac{\pi \sqrt{P_{c}}}{2\sqrt{\mathrm{nC}}}$$

In Equation (5), l is the twisting length of two fibers. The coupled power increases by increasing the length of the twisting of two fibers. In taper and fuse technology, the coupling power ratio is estimated to be constant during manufacture [48]. The coupling power can be changed if there is any disturbance in the coupling region. According to the couple-mode theory, the disturbance is forbidden in the taper and fuse method [48]. However, the disruption is imposed on the coupling part in terms of bending in this work. When the incident light propagates along the bending portion of the fiber, some refraction and tunnel light will be produced because the angle of the incident light exceeds the critical angle. In this case, some light will be refracted from the first fiber and coupled with adjacent fibers. For Equation (2), the coupling coefficient between the two fibers can be determined. The coupled power in the second fiber is measured through a power meter and calibrated at different rotational positions.

## 3. Experimental Work and Results

Figure 1 shows the experimental setup of the design structure. The fibers used in the test were composed of polymethyl methacrylate resin and fluorinated polymer, with core and cladding diameters of 980 μm and 1 mm. Furthermore, the refractive index of the core was 1.49. Because the core diameter is large and the cladding area is small, it is advantageous to produce significant bending loss and tight-side coupling [49]. The robot drags the twisted fiber, which causes the change in the macro-bend radius.

The experimental platform was also developed to integrate the rotational sensor into the robot. The experimental platform’s configuration contains a linear and circular screw and driver unit to operate the robot. The resolution of the robot was 1.8°, which relies on the stepper motor. At one complete rotation from 0° to 360°, the linear screw moved from 0 mm to 120 mm. Next, a linear screw was attached to the circular screw, and the circular screw was fixed with the robot. One end of the twisted fiber was fixed on the linear screw. Thus, the experimental configuration converts circular motion into linear motion whenever the robot moves. The robot movement drags the twisted fiber that causes a change into a bending radius.

**Figure 2:**
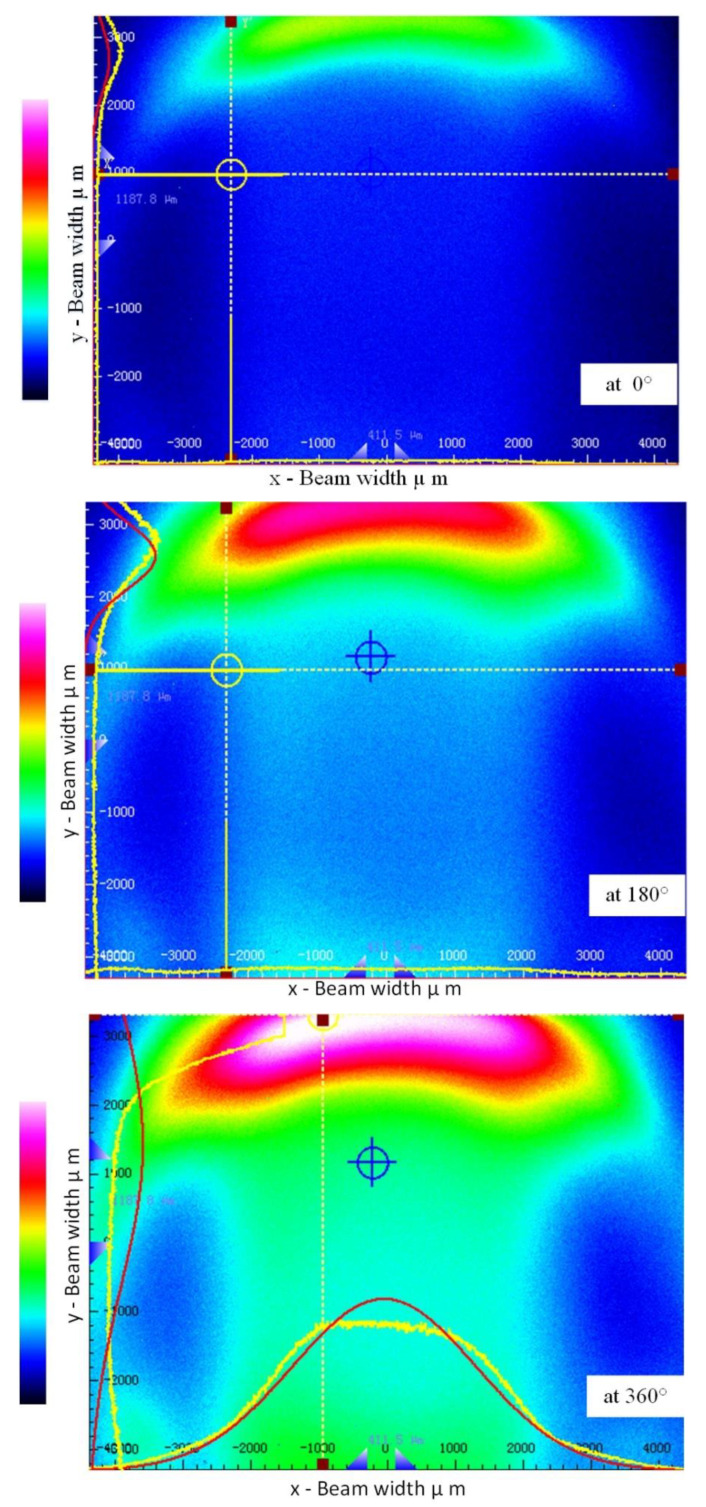
The response of the coupled intensity in the second fiber using a beam quality analyzer.

Initially, we analyzed the side-coupling response in the second fiber using the beam quality analyzer (Thorlabs BC106N-VIS/M) to validate that the radiation loss was being coupled in the second fiber. We observed the light intensity of the second fiber at different rotational positions (i.e., 0°, 180°, and 360°) on the beam quality analyzer. Figure 2 shows the light intensity distribution taken from the fiber’s forward-end. In Figure 2, the center corresponds to the fiber’s core, while the edge corresponds to the fiber cladding. In the illuminating fiber, the core is the main source of mode field energy as the light energy is confined in the core. The bending in an illuminating fiber causes the light intensity to gradually reduce from the center of the core to the cladding environment. Different rotational positions result in a change in bending radius, which creates a bending loss. According to Figure 2, the light intensity increased consistently with an increase in the bend radius of the second fiber. When the rotation starts from 0° to 360°, it constantly changes the bending radius and the bend loss is also raised proportionally. The bending loss theory can estimate the lost power in the illuminating fiber [50]. However, our concern was to couple the lost power in the second fiber, which is why the beam quality analyzer was utilized to know if the lost power is being coupled. It should be noted that the test was conducted to validate that the intensity varied with different rotational positions. As shown in Figure 2, the coupled bending loss in the second fiber usually propagated in the cladding region. This shows that light intensity is concentrated in the cladding region due to the side-coupling effect. The second fiber could be side-polished to couple into the core region, but scattering and fluctuation occurred in the fiber due to the side-polishing technique [51]. However, the intensity varied in twisted structures without any side-polishing or tapering techniques. Our aim was not to confine the light in the core region. Thus, a power meter was required to measure the coupled intensity in the second fiber.

**Figure 3:**
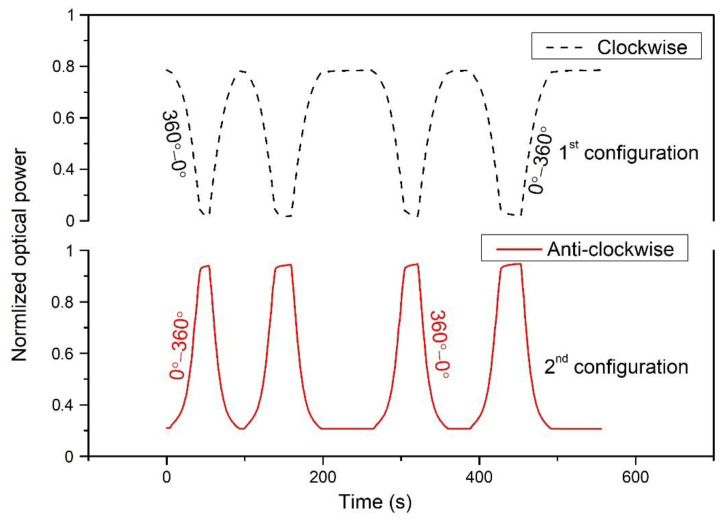
The clock and anti-clockwise rotation of the sensor over time.

In Figure 3, the coupling power was measured using the power meter and achieved results that both clockwise and anti-clockwise rotations were operated in the specified periods. A consistent rotational movement of about 12° per second was applied, and 30 samples were measured on a single rotation. The change in bending radius was the main cause of the sensor’s sensing. The sensor configuration depended on the bend radius position and two scenarios could be possible: the bend-radius continuously increases or decreases as the robot moves (1st configuration for clockwise, and 2nd configuration for anti-clockwise). The clockwise (360° to 0°) and anti-clockwise (0° to 360°) rotation depends on the bending configuration. It was noticed from Figure 2 that there the minimum intensity was coupled at 0°, and the maximum intensity was coupled at 360° when the bending radius gradually decreased. This could be vice-versa if the bending configuration is reversed, and the bending radius will be increased from the maximum to minimum. Figure 3 shows the sensor response when the robot initially moves in clockwise and anti-clockwise directions. In both curves, the initial power coupling showed different responses: one started from a higher optical power and the other started from a lower optical power. This is because of the initial bending configurations, which were set for rotational direction. It is worth noting that the initial rotational direction depends on the operation. If starting with a clockwise operation, then anti-clockwise rotation has to operate to reverse back to the initial point and vice-versa. However, we configured a maximum bend radius when the robot started to move in a clockwise direction while the initial power coupling was the maximum and power decreased with rotation. In contrast, the bending radius was the minimum during anti-clockwise rotation.

In Figure 3, as the rotation shifted from 0°–360°, the curve began to rise due to the increase in coupling power. Throughout the curve’s rise, the twisted fiber’s bending radius decreased. At 360°, the coupling power became constant and then rotated in reverse, starting from 360° to 0°. The rising curve indicates the increase in the coupling power, while the decreasing curve indicates the decrease in the coupling power. Since the bend radius was kept to a minimum, the start rotation was anti-clockwise. After a 360°change, the bending radius was the largest, and the illuminating fiber’s radiation power loss was significantly higher. At 360°, the coupling power changed clockwise during this period with the decrease in the bending radius.

**Figure 4:**
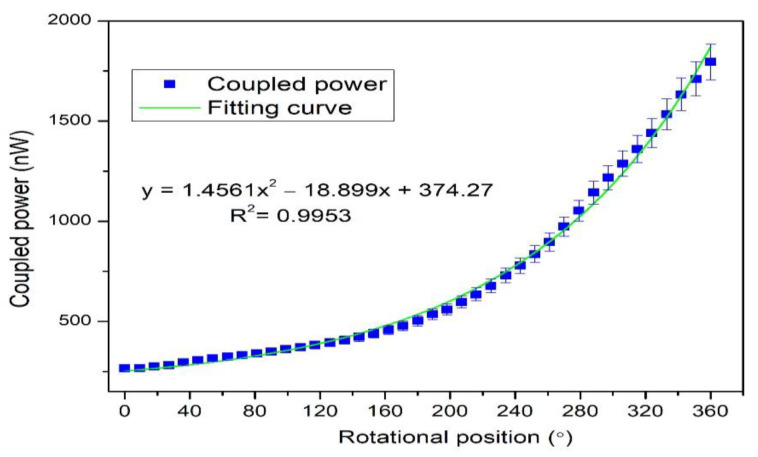
The anti-clockwise measurement of the rotational sensor.

**Figure 5:**
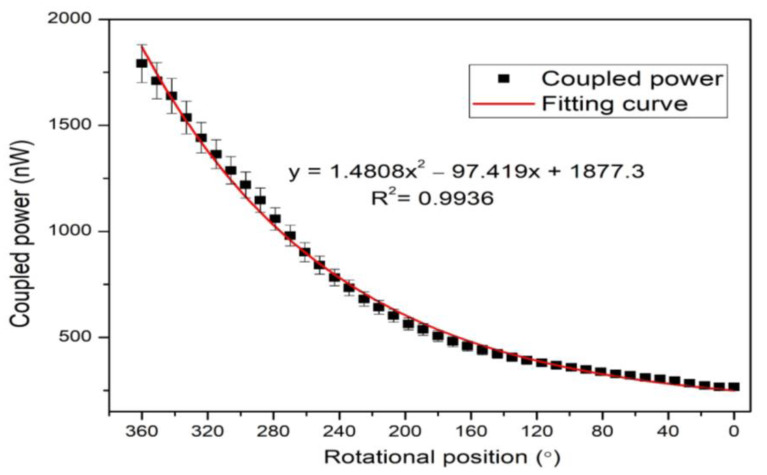
The clockwise measurement of the rotational sensor.

The clockwise and anti-clockwise rotational positions were measured in the experiment (see Figure 4 and Figure 5). When the robot’s rotational position changed gradually, the circular screw began to start. Therefore, the twisted fiber’s bending radius gradually changed, reducing the influence on the twisting bending radius. The variation occurred in the bending loss concerning the bending radius, which began to increase. The lost power from the illuminating fiber radiated in the environment, while a specific amount of lost power was coupled with the neighboring fiber. The lost power was efficiently coupled in the second fiber as the second fiber (SK-40) had a large core diameter. In the anti-clockwise direction, the rotation varied from 0°–360°, and the bending radius decreased. In the clockwise rotation, the bending radius increased, and the rotation varied from 360° to 0°. It was noticed from the curve that the coupled power was linear up to 180°; then more power was coupled due to a sharp increase in the bending loss.

**Figure 6:**
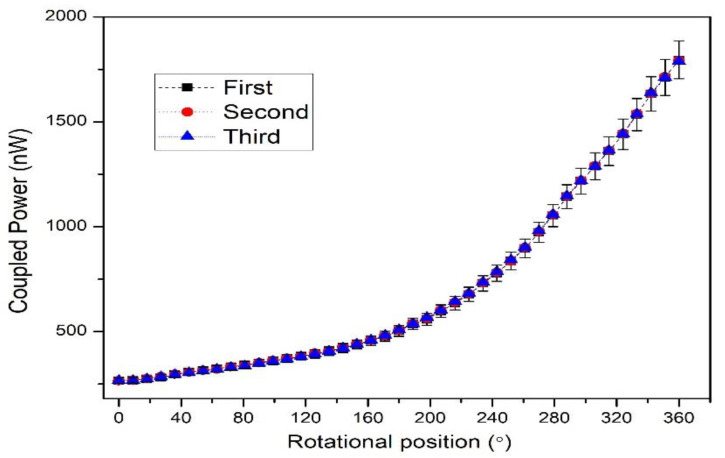
The repeatability response of the rotational sensor.

**Figure 7:**
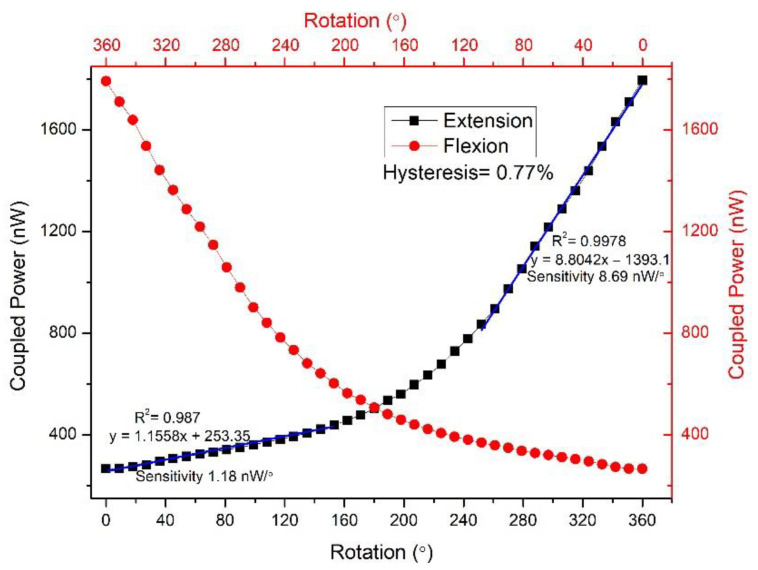
The rotational sensor response with optimized sensitivity and hysteresis.

The rotational step change can be varied by applying different input conditions to the drive unit. Thus, in the experimental condition, a smaller step-change could also be achieved such as 3.6°. The 100 samples were measured in the 3.6° step-change state. The repeatability of the proposed rotational sensor was also tested. The anti-clockwise rotation was performed three times, and the obtained data are shown in Figure 6. Each test’s measured coupling power had a good consistency, and R square was about equal to 1. We used polynomial degree 2 for the fitting curve as the power coupling sharply increased after a certain rotational position due to a sharp increase in the bending loss. However, this quadratic curve can be divided into two linear quasi-distributed curves. The sensitivity between two quasi-distributed curves is shown in Figure 7, where the sensitivity was calculated as the difference between the final and initial coupling response divided by the change in rotational position. Both the initial and final coupling responses were acquired in nanowatts (nW) and the rotational position in degrees (°) (see Equation (6)) [52].

(6) $$\mathrm{Sensitivity}\;\left(\mathrm{nW}/{}^{\circ}\right)=\frac{Po(\theta_{f})-Po(\theta_{i})}{\Delta \theta }\;$$

where $Po(\theta_{f})$ is the final power coupling and $Po(\theta_{i})$ is the initial power coupling. The sensitivity between 0° and 163° was 1.18 nW/°, and 243°–360° was 8.69 nW/°. The second region had a higher sensitivity because bending loss occurred sharply after a threshold point. Furthermore, the sensitivity can increase if the power between two fibers is coupled. As shown in Figure 2, the coupled power in the second fiber penetrated the cladding region due to the side-coupling effect [49]. The power can penetrate into thee core region if the cladding region is removed, but it will be a destructive method that might damage the fiber and increase scattering. An alternative method could be the use of agarose gel [53,54]. Normally, the refractive index is 1 due to the air medium between two twisted fibers. Due to bending loss, some of the power that refracts out of an air-medium can re-refract and couple in the fiber by using agarose gel. Equation (7) shows the calculation for hysteresis. The hysteresis ratio can be calculated as the vertical difference between extension and flexion over the difference between the initial and final points.

(7) $$\mathrm{Hysteresis}\;\left(\%\right)=\frac{Y_{en}-Y_{fl}}{Y_{max}-Y_{min}}\times 100\%\;$$

where $Y_{en}-Y_{fl}$ is the difference between the flexion and extension curve on a designated point and $Y_{max}-Y_{min}$ is the difference between the maximum and minimum points. According to the calculation, the hysteresis was less than 1 percent, which was 0.77 %.

**Figure 8:**
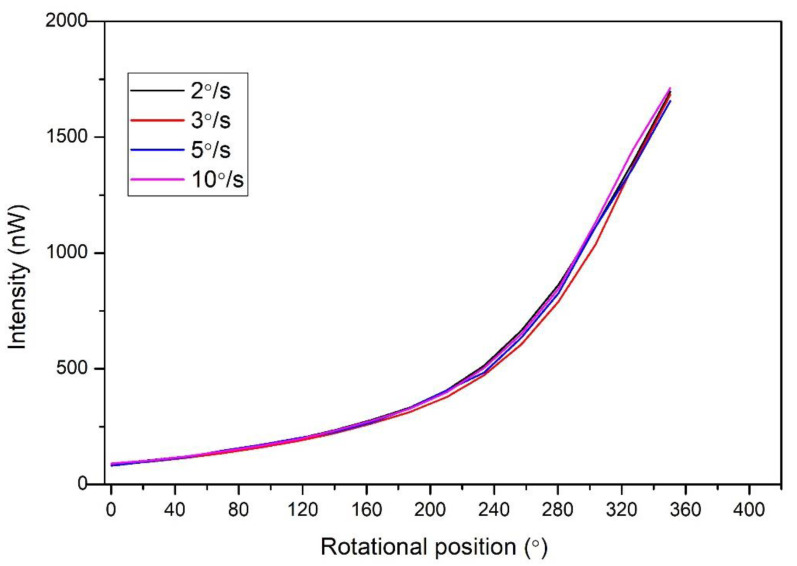
Dynamic response of the rotational sensor.

The results as above-mentioned in Figure 4 and Figure 5 were measured as a constant measurement to analyze the coupled intensity. It was found that the macro bend radius became smaller while the received intensity increased, which gradually caused a decrease in the macro bend radius. After that, the different rotational speed was carried out to obtain the dynamic response of the sensor. The robot was operated through the controller and set as dynamic motion. When the controller’s start button is pressed, the robot’s motor starts with a constant speed from 0 degrees and stops at 360 degrees. The four different speeds were set in a control unit that controls the rpm of the robot’s motor. The sensor response was conducted at 2°/s, 3°/s, 5°/s, and 10°/s rotational speeds. The response is shown in Figure 8. It can be seen from the results that four dynamic motions were carried out where the sensor had a similar output. The received intensity did not change with a dynamic response compared to the static measurement.

**Figure 9:**
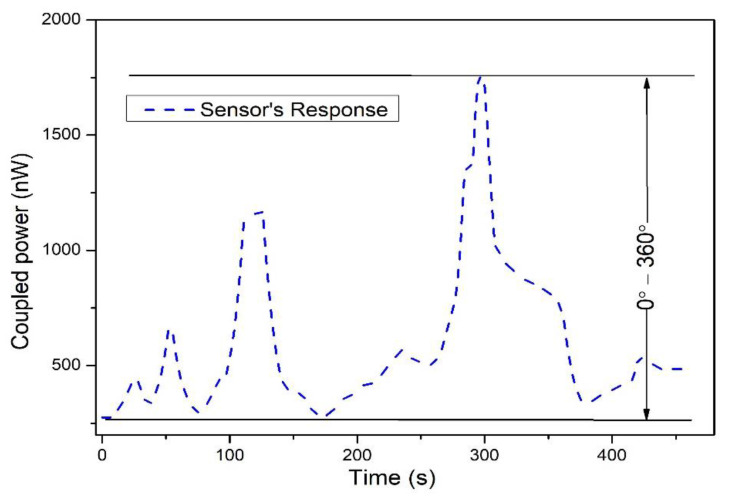
Sensor response to randomly operated rotational motion over time.

In all of the previous results, a full rotation cycle was applied to the robot to measure the coupled power response versus the rotational position. There are two options to operate the robot automatically or manually from the driver unit. An automatic function was used in which the robot was instructed to stop automatically after reaching 360° of rotational position. The rotation started after pressing the ON button and stopped automatically at 360°, the same as for reverse motion. However, an instant response was conducted on the robot movement assessment (rotation can increase or decrease instantly). The sensor configuration was set to increase the bending radius, and the driver unit was configured to either press button-1 or button-2 for forward and reverse movement (clockwise or anti-clockwise movement). The response of the integrated sensor on a robot is shown in Figure 9. The manual function was used for the results presented in Figure 9, in which movement depended on pressing the button in the driver unit. In this, pressing and holding button-1, the robot moves forward (clockwise) and stops when releasing and holding button-1. Likewise, button-2 is used to move reverse (anti-clockwise). In the different time intervals, button-1 and button-2 were pressed; we refer to this movement as random movement of the robot.

The rise in the coupling indicates that the rotation position increased, and the falling curve shows that rotation decreased. The sensor had good consistency as randomly rotational motions were operated between 0° and 360°. A comparison of the rotational sensor is shown in Table 3. It is worth noting that the sensor response is affected by humidity as two fibers are twisted together. As stated earlier, the fibers were coated with a black tube after twisting. The black tube not only shields the external light, but also prevents the effect of humidity.

Moreover, the experiments were conducted in the ambient temperature of the room. According to the datasheet, the temperature range of Mitsubishi’s SK-40 fiber is 110 °C. Therefore, sensor calibrations will be required at different temperatures. The proposed dynamic rotational sensor has a straightforward, simple design and is easily implemented on the robot structure. Moreover, this sensor can also be easily integrated into real applications in the robotic field. A simple LED will connect with POFs as a light source. To detect light intensity, a simple photodetector will be required. This can extend to three degrees of freedom where three rotational motions can be measured. However, additional tests can also be conducted in future studies such as the rotational sensor’s multiplexing response, temperature, and humidity effect.

## 4. Conclusions

A simple and straightforward polymer fiber-optic dynamic rotational sensor for robot movement assessment was proposed in this work, relying on the intensity variation. The sensing phenomenon was based on side-coupling between two fibers, where two POFs were twisted and bent. The sensor structure consisted of a light source, power meter, stepper motor, and two POFs. Both fibers were bent after twisting. The power-variation at the detector was associated with a different rotational position. The power-variation was caused by the macro-bend loss in the fiber that drags with the robots. The experiment tested the robot in clockwise rotational, anti-clockwise rotational, and dynamic motion. A full cycle rotational measurement was achieved (i.e., 0–360°) and in dynamic response, different rotational speeds (2°/s, 3°/s, 5°/s, and 10°/s) were conducted. Moreover, the sensor had low hysteresis loss (0.77%) and had good sensitivity of about 8.69 nW/°. It was found that the sensor can work in both static and dynamic conditions. The designed sensor is easy to fabricate, handle, and integrate on a robotic structure and a cascading method can be used in this structure to integrate three sensors on a single transmission fiber and used for three degrees of freedom (3-DOF). In future work, multiple rotational sensors can be achieved on a single fiber using the cascading technique.

## Figures and Tables

**Table 1:** The characteristic parameter of the Mitsubishi SK-40 PMMA fiber.

Parameters	Values
Core material	Polymethyl methacrylate
Clad material	Fluoropolymer
Refractive Index Profile	Step refractive index
Numerical aperture	0.5
Transmission attenuation	150 (dB/km)
Refractive Index	1.49
Core diameter	980 µm
Cladding diameter	1 mm

**Table 2:** Sensor parameters.

Parameter	Values
Fiber length	1 m
Twisting length	10 cm
Twisting region	From 40 cm to 50 cm
Initial bending radius	4 cm

**Table 3:** Comparison of rotational sensor.

Ref.	Method	Range	Sensitivity	Fiber type
[22]	FBG sensor	0–150°	0.012 nm/°	FBG
[38]	Two abrupt-tapers modal interferometer	0.0575° and 0.075°	601.8 nm/°	Single-mode optical fiber
[39]	Interferometer	0°–90°	55.67 pm/◦	PCF
[40]	FBG sensor	−21°–21°	0.743 nm/°	FBG
[41]	Coherence interferometry	0–360°	6.2 fringe/°	Seven core fiber
[42]	Moiré pattern	0°–30°	2.1 µW/°	POF
[44]	Intensity variation	0°–90°	20.9 mV/◦	POF
In this work	Intensity variation	0°–360°	1.18 nW/° (0–163°) 8.69 nW/° (243–360°)	POF

## Data Availability

The data will be available on demand.
